# Supplementary figures and images for: Homologous chromosomes are stably conjoined for Drosophila male meiosis I by SUM, a multimerized protein assembly with modules for DNA-binding and for separase-mediated dissociation co-opted from cohesin
Source: PLoS Genet. 2022 Dec 8;18(12):e1010547. doi: 10.1371/journal.pgen.1010547 (PMC9767379; doi:10.1371/journal.pgen.1010547)

**A**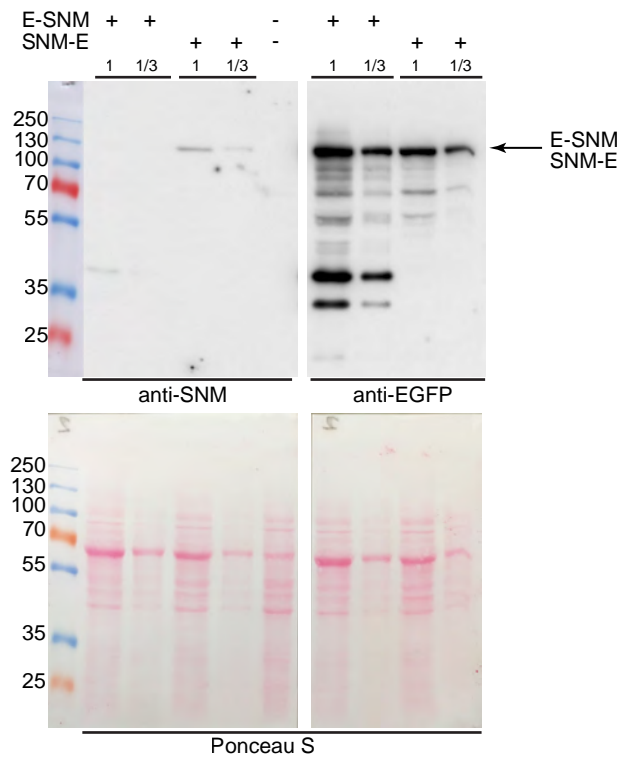**C**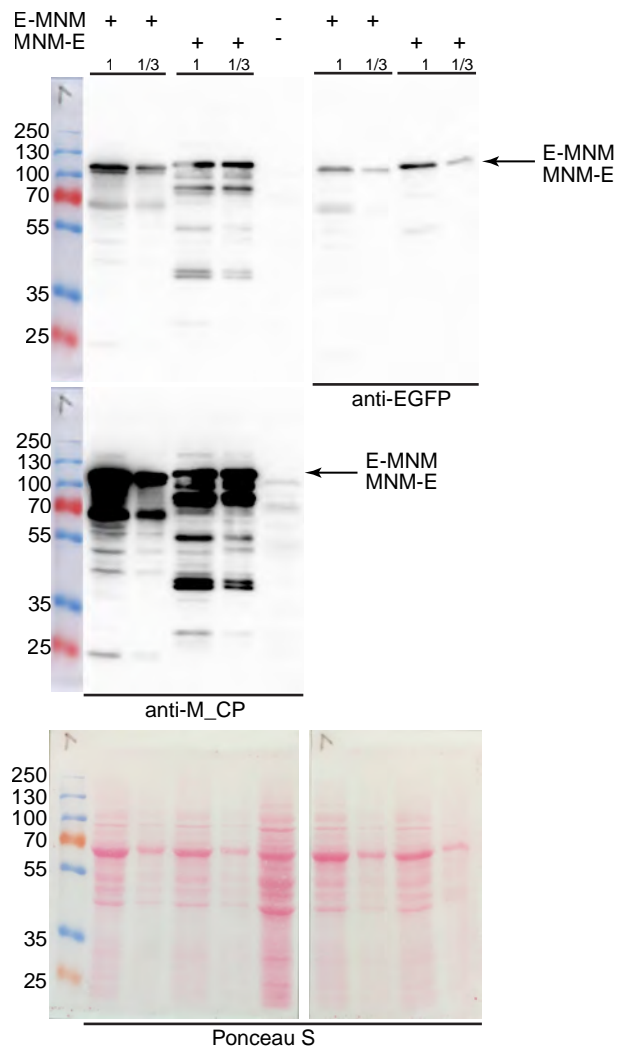**B**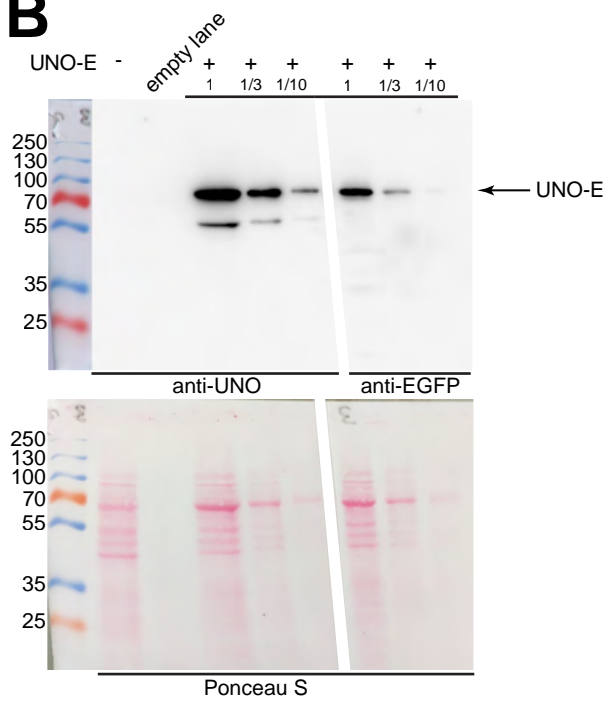

Supplement: S1 Fig — (A-C) Total extracts of S2R+ cells, either untransfected or transfected for transient expression of EGFP-SNM (E-SNM), SNM-EGFP (SNM-E), UNO-EGFP (UNO-E), EGFP-MNM or MNM-EGFP (MNM-E) were analyzed by immunoblotting with the indicated antibodies. Prestained marker proteins with the indicated molecular weights (kDa) are displayed on the left. Relative amounts of extract loaded are indicated on top. Ponceau S staining of the membrane after protein transfer for control of loading is presented below the immunoblots. (A) A band indicating expression of endogenous SNM is not detected by anti-SNM, in contrast to transiently expressed SNM-EGFP, confirming antibody functionality. (B) A band indicating expression of endogenous UNO is not detected by anti-UNO, in contrast to transiently expressed UNO-EGFP, confirming antibody functionality. (C) An antibody (anti-M_CP) against the common part present in MNM and the other isoforms expressed from the mod(mdg4) locus detects bands in untransfected S2R+ cells that are much weaker than those reflecting transiently expressed MNM-EGFP. The anti-M_CP immunoblot is shown after short (top) and long exposure (bottom) to reveal these weak bands. (PDF) [file pgen.1010547.s001.pdf]

**A**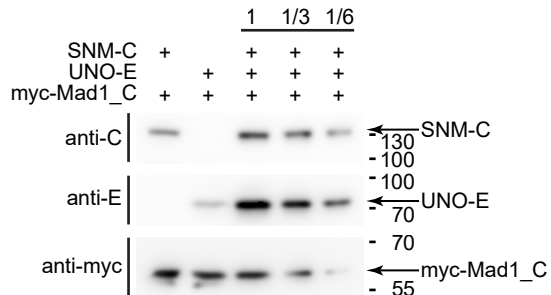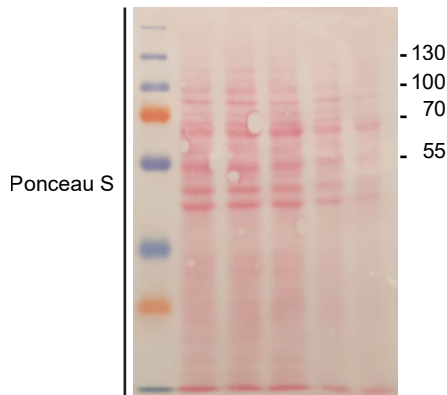**B**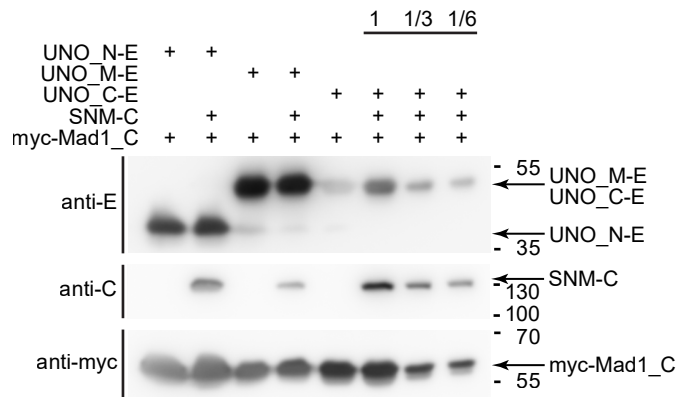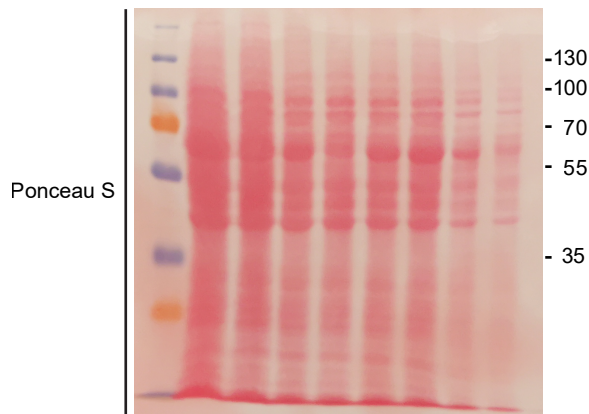

Supplement: S3 Fig — Co-expression of SNM with UNO or with the UNO_C fragment results in higher expression levels in comparison to the individual expression of these proteins. Levels of tagged proteins resulting after transient expression of UNO (A) or UNO fragments (B) together with the indicated proteins in S2R+ cells were analyzed by immunoblotting. SNM was tagged with mCherry (SNM-C). UNO (A) and the fragments UNO_N, UNO_M and UNO_C (B) were tagged with EGFP (UNO-E, UNO_N-E, UNO_M-E and UNO_C-E). A plasmid for expression of a C-terminal fragment of Mad1 tagged with a myc-epitope (myc-Mad1_C) was co-transfected for comparison of transfection efficiencies. Total cell extracts were resolved and analyzed with anti-mCherry (anti-C), anti-EGFP (anti-E), and anti-myc (anti-myc). Relative amounts of extract loaded are indicated on top. Ponceau S staining of the membrane after protein transfer for control of loading is presented below the immunoblots. The positions of prestained marker proteins with the indicated molecular weights (kDa) and of the bands representing the indicated proteins of interest are displayed on the right. (PDF) [file pgen.1010547.s003.pdf]

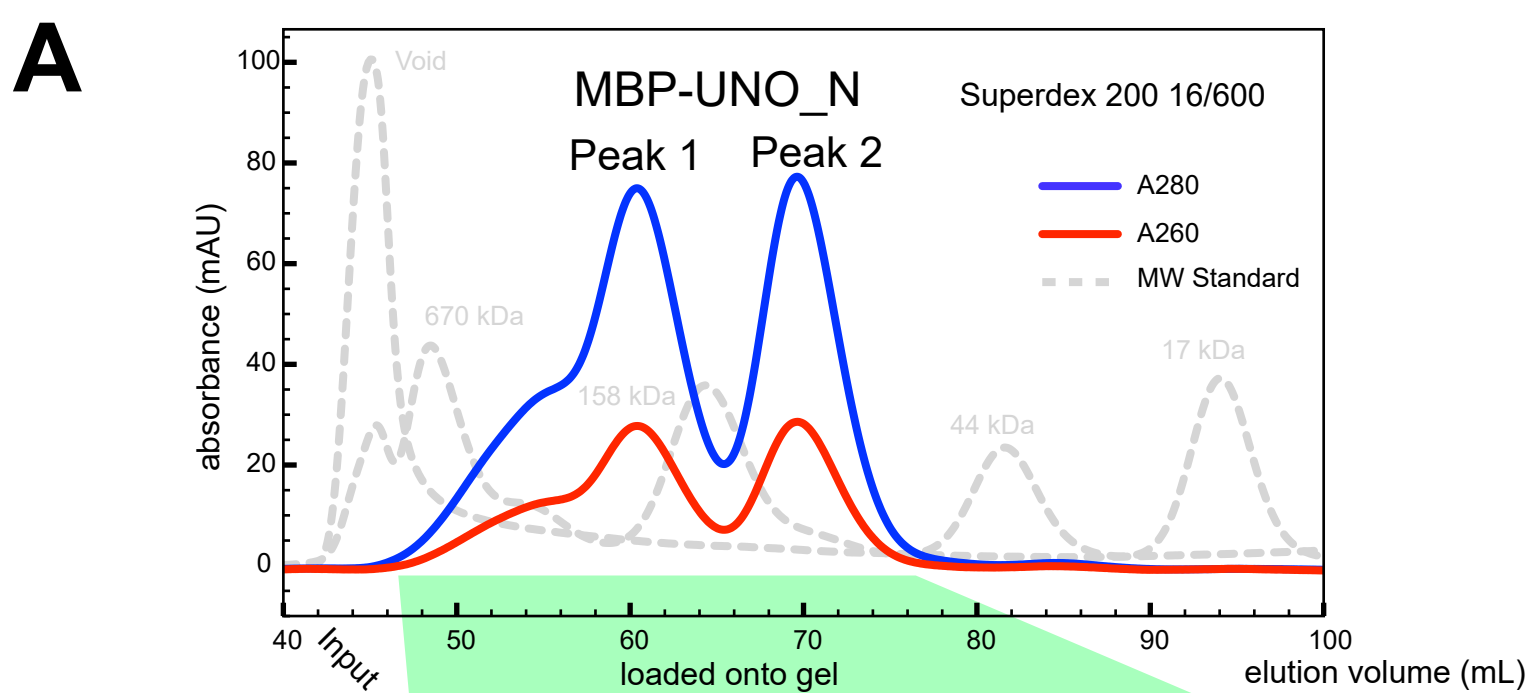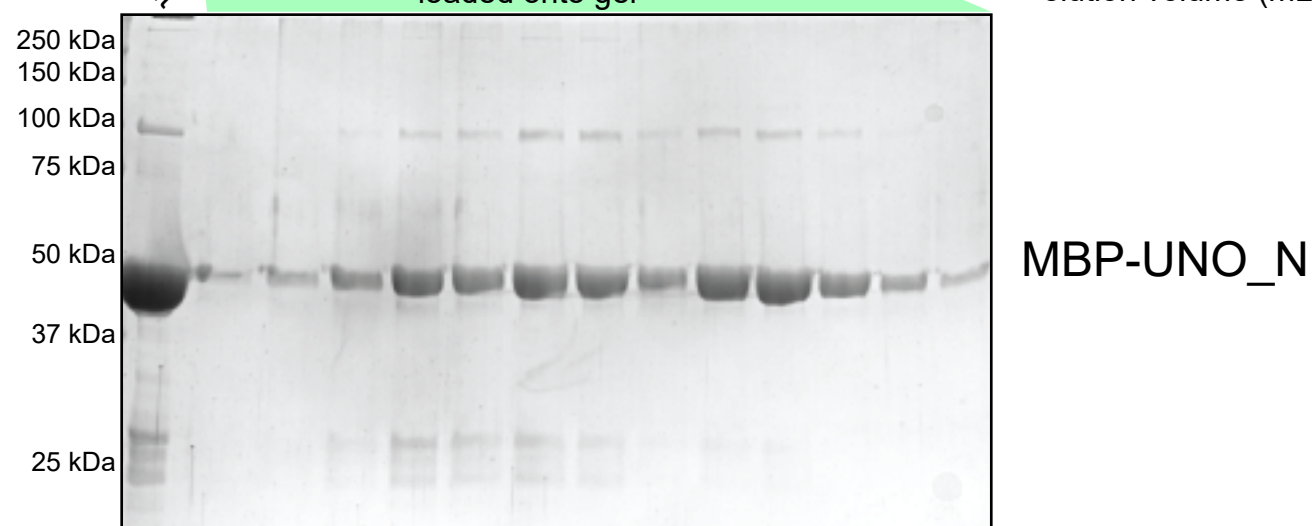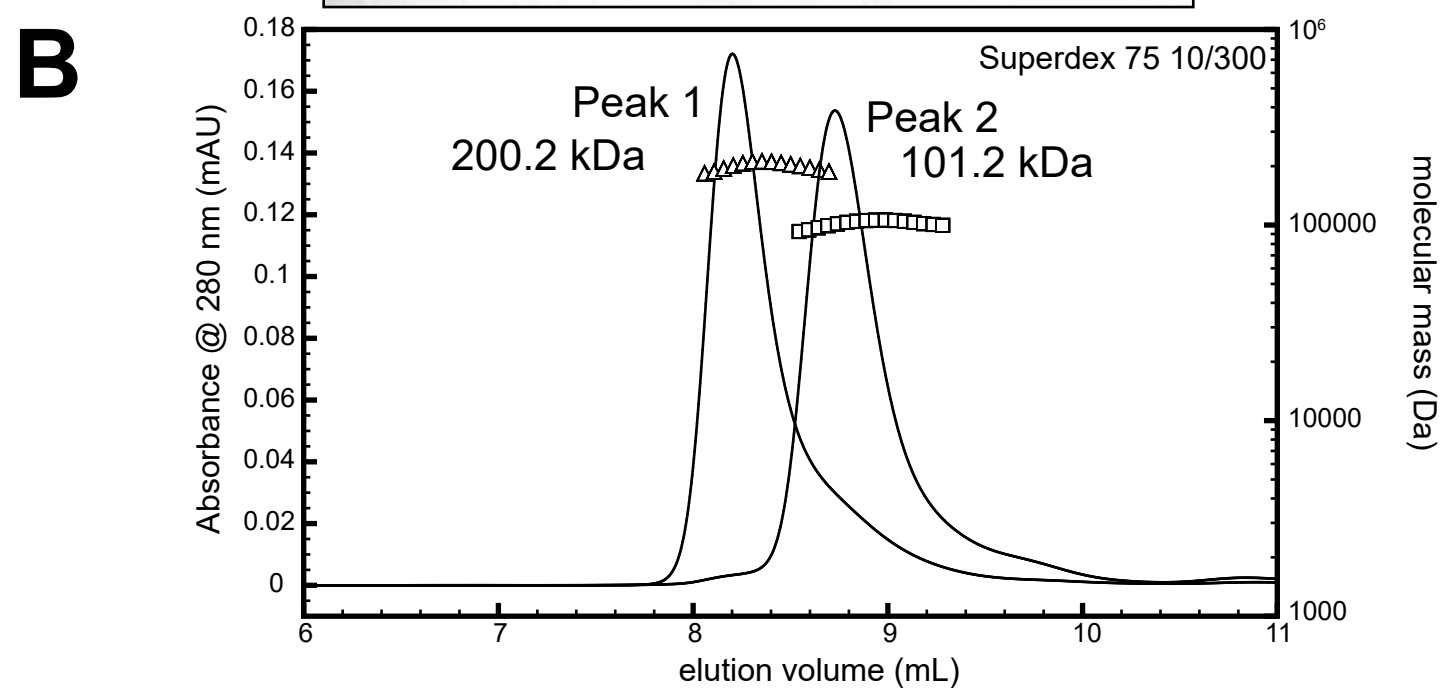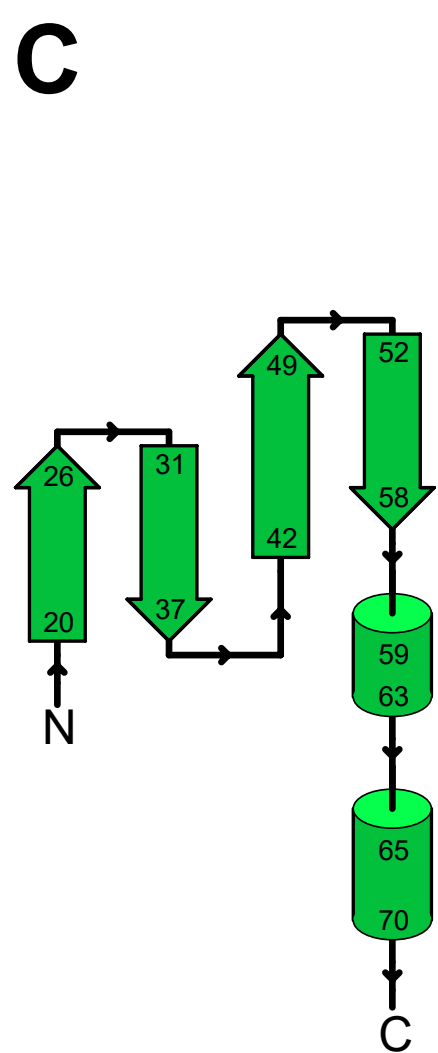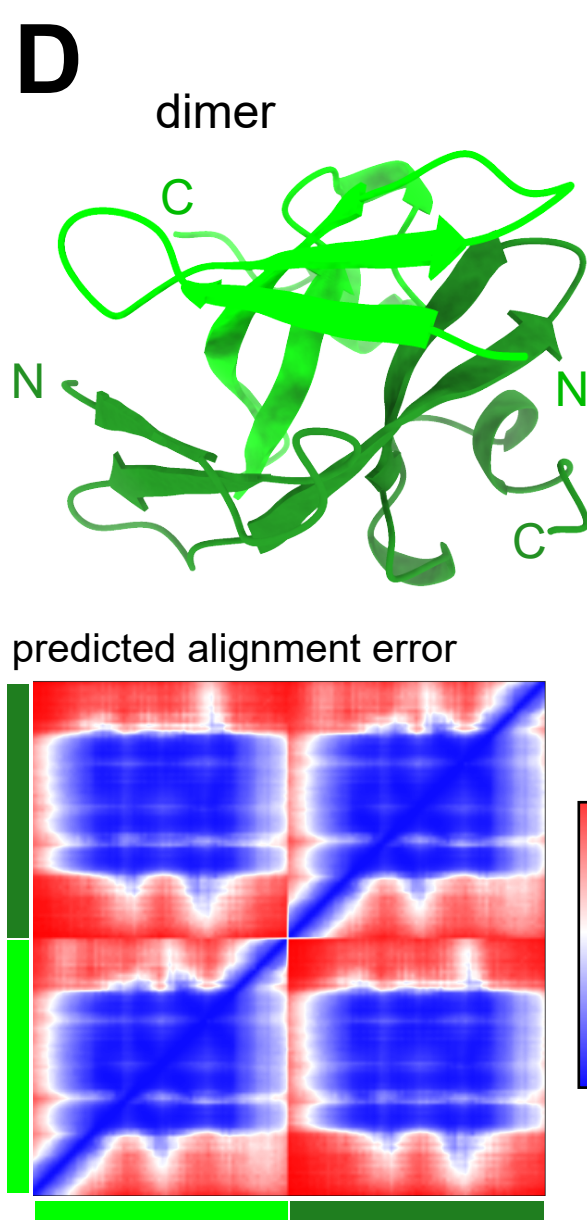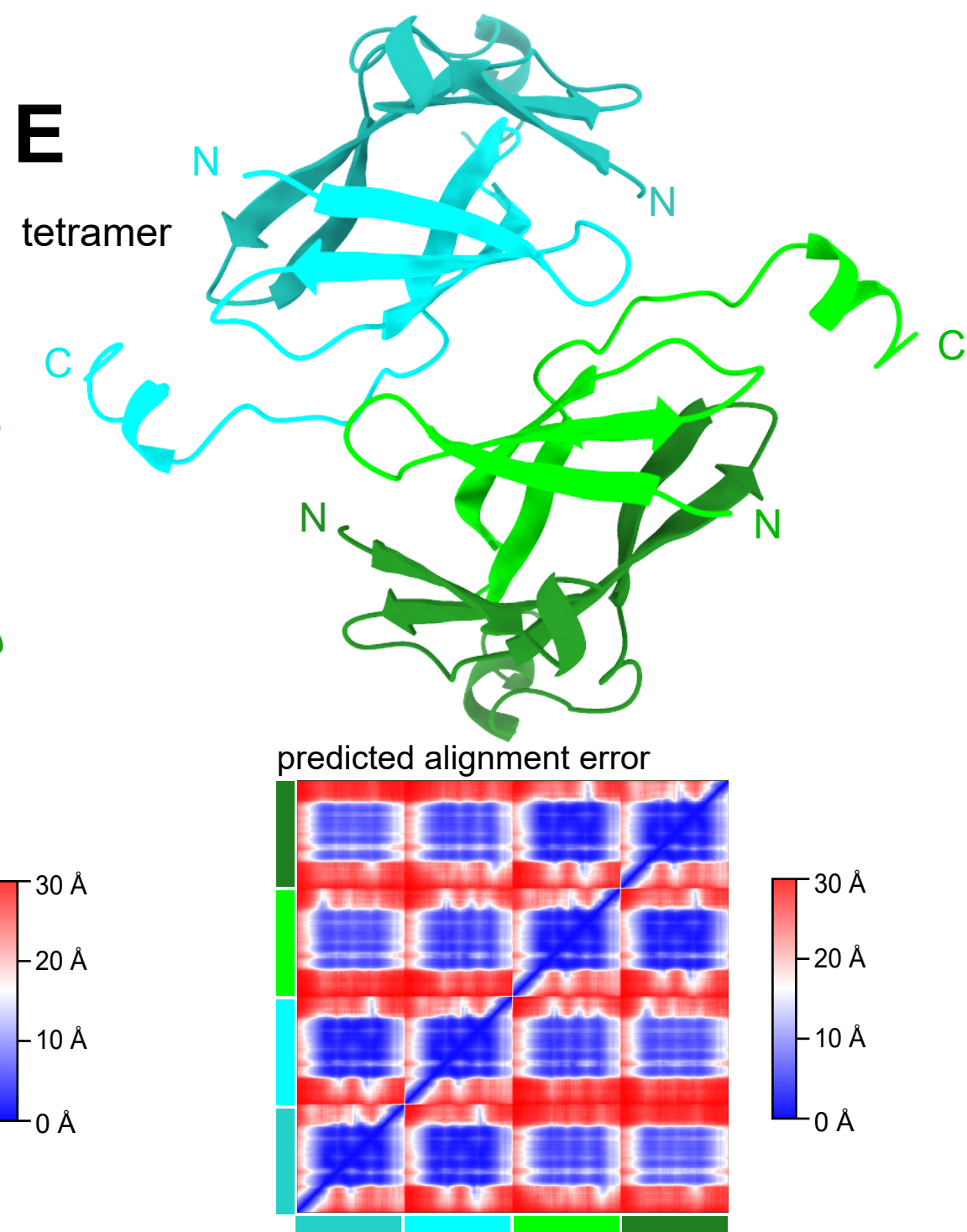

Supplement: S4 Fig — (A) Final purification step of UNO_N. The SEC profile revealed two peaks (Peak 1 and Peak 2), both containing only UNO_N according to SDS-PAGE and Coomassie staining. (B) SEC-MALS of the UNO_N complexes in Peak 1 and Peak 2 resulted in the indicated molecular masses. (C) Topology map of a UNO_N monomer. (D) AF2 model of the UNO_N dimer with predicted alignment error shown below. (E) AF2 model of the UNO_N tetramer with predicted alignment error shown below. (PDF) [file pgen.1010547.s004.pdf]

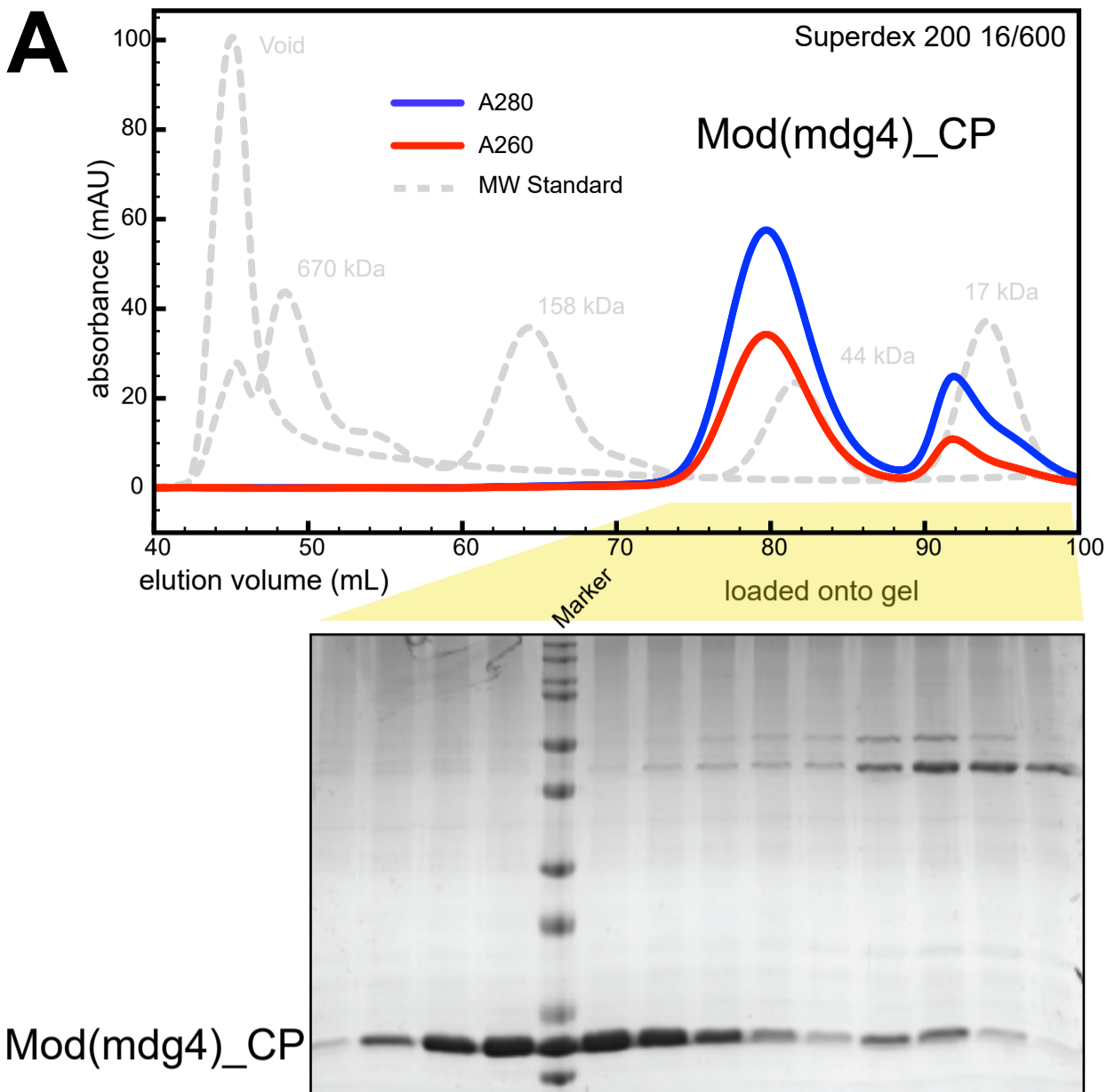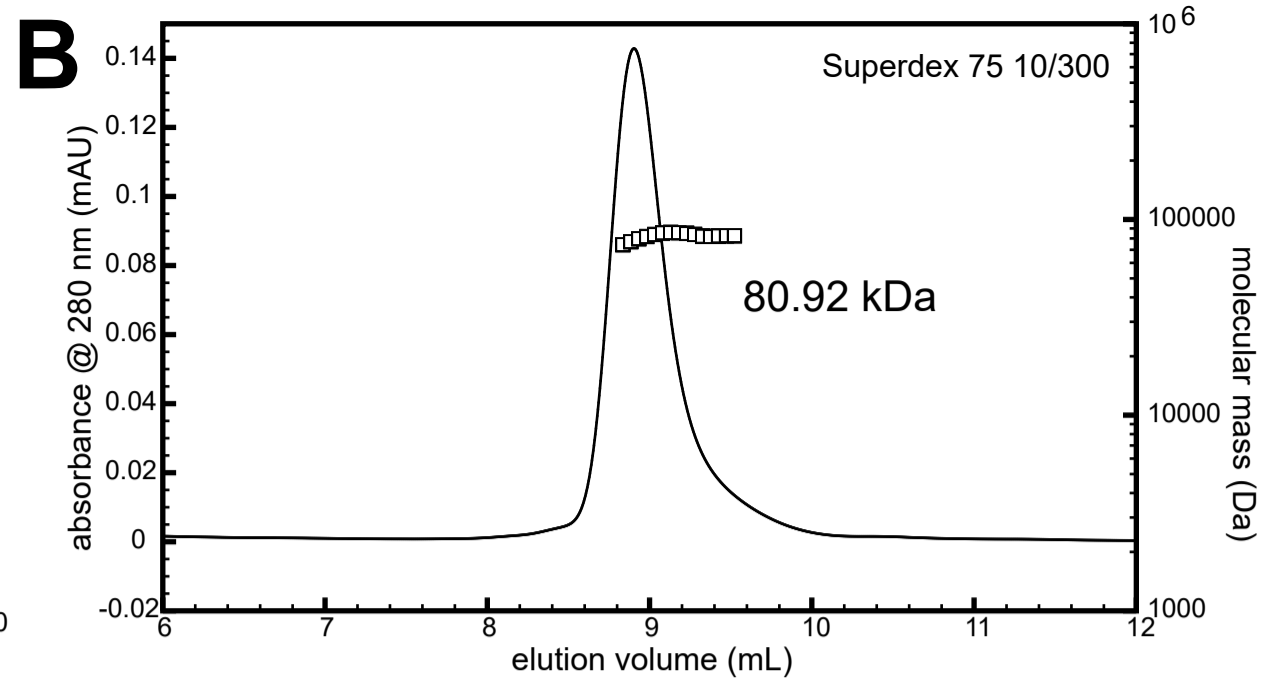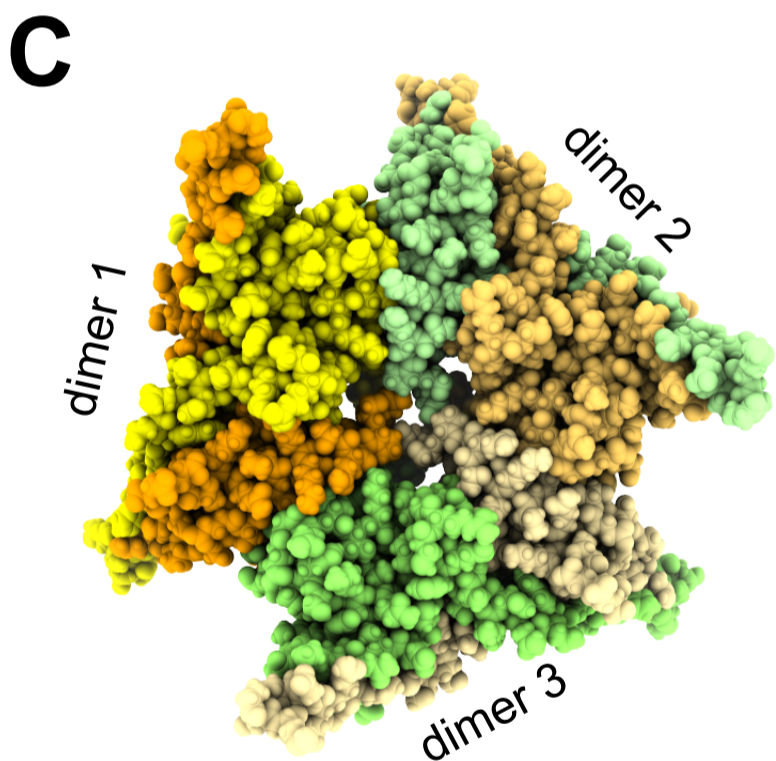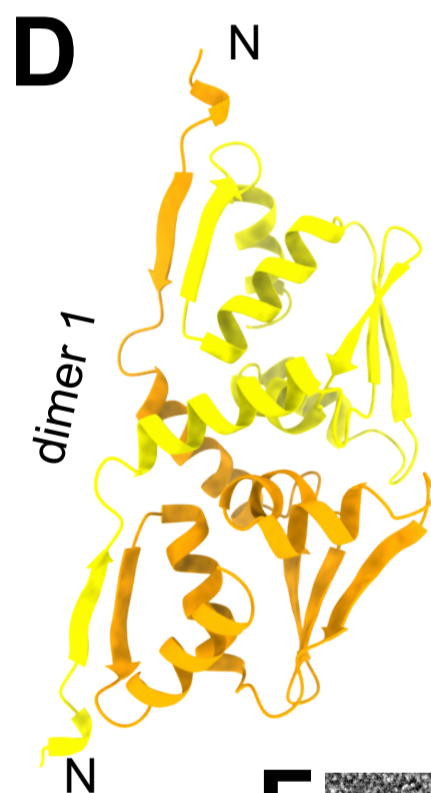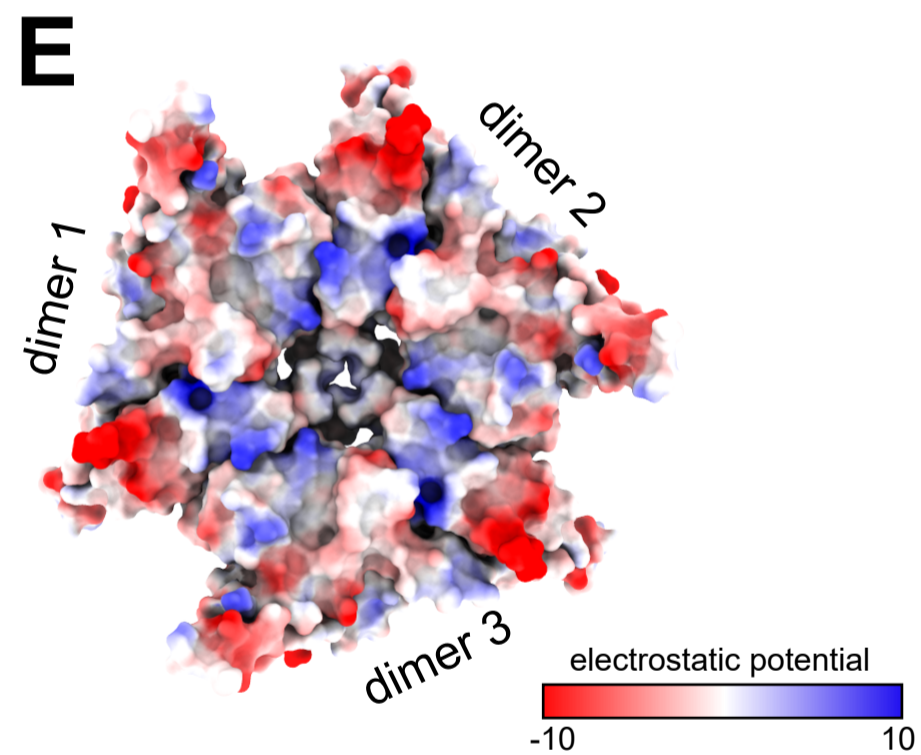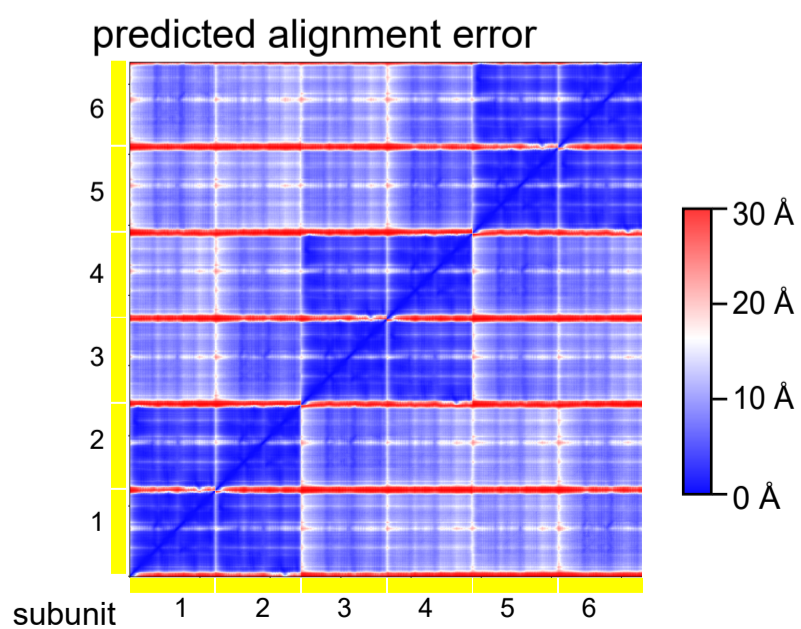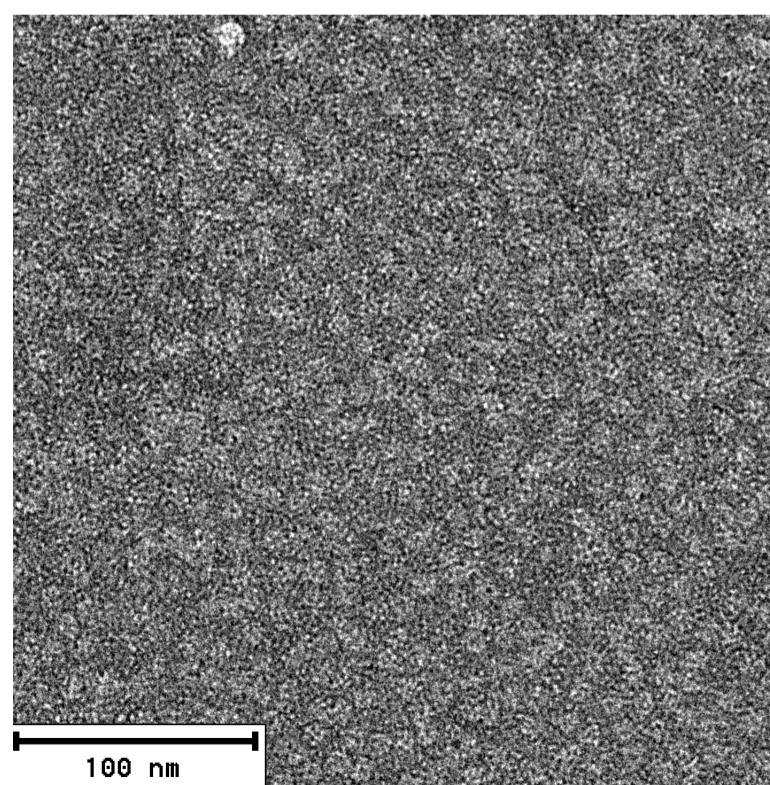

Supplement: S5 Fig — (A) Final purification step of Mod(mdg4)_CP, the N-terminal part of MNM, which is also present in other Mod(mdg4) protein isoforms. Analysis by SDS-PAGE and Coomassie staining indicates that the peak obtained by SEC contains pure Mod(mdg4)_CP in a multimerized form. (B) Molecular mass determination of purified Mod(mdg4)_CP by SEC-MALS resulted in a value of 80.92 kDa, consistent with a hexamer formation. (C) AF2 model of Mod(mdg4)_CP hexamer with predicted alignment error shown below. (D) Ribbon diagram of one of the three Mod(mdg4)_CP dimers that form the hexamer according to the AF2 model. (E) Surface electrostatics on the Mod(mdg4)_CP hexamer showing a lack of obvious DNA-binding regions. (F) NS-EM image of the Mod(mdg4)_CP hexameric rings. (PDF) [file pgen.1010547.s005.pdf]

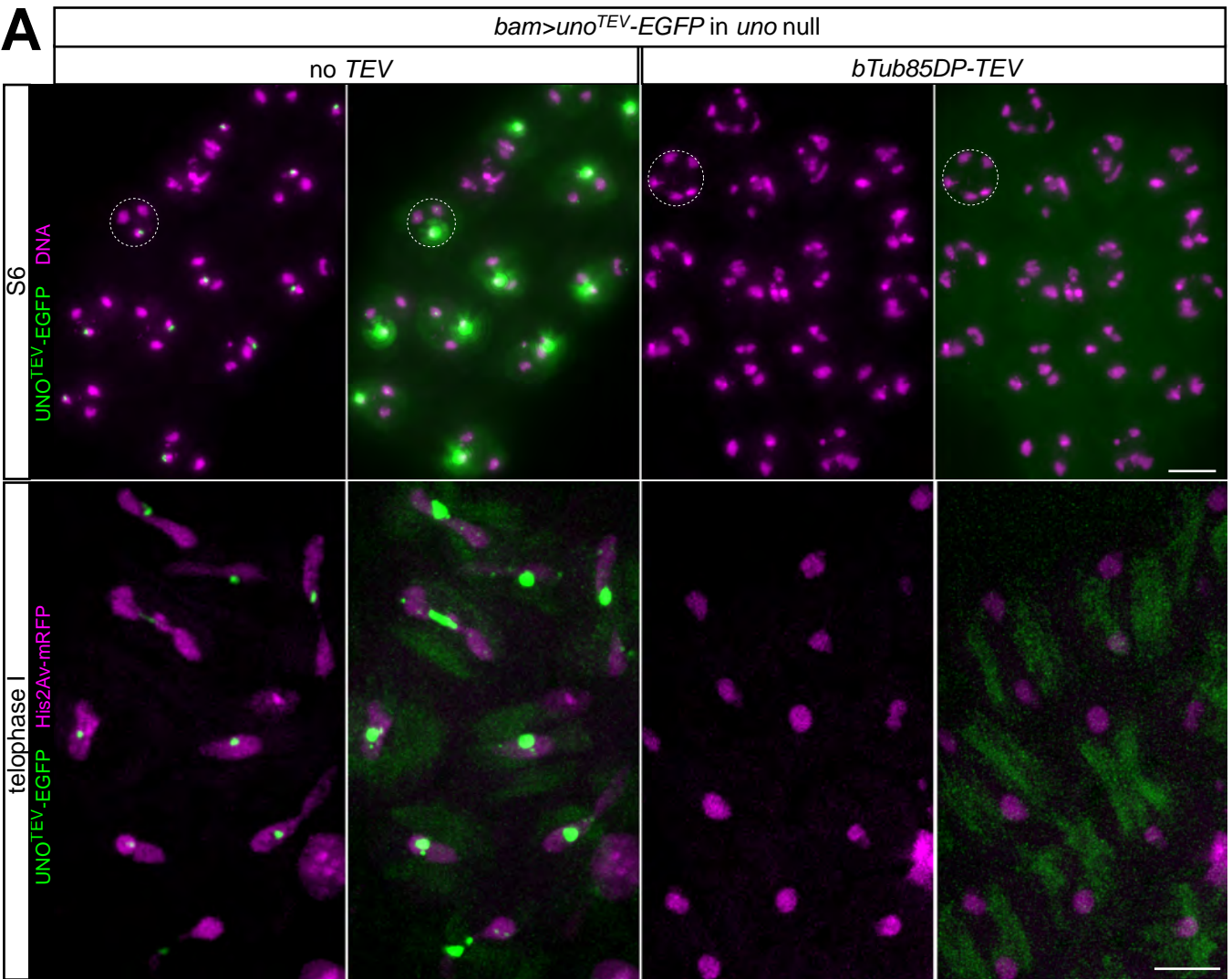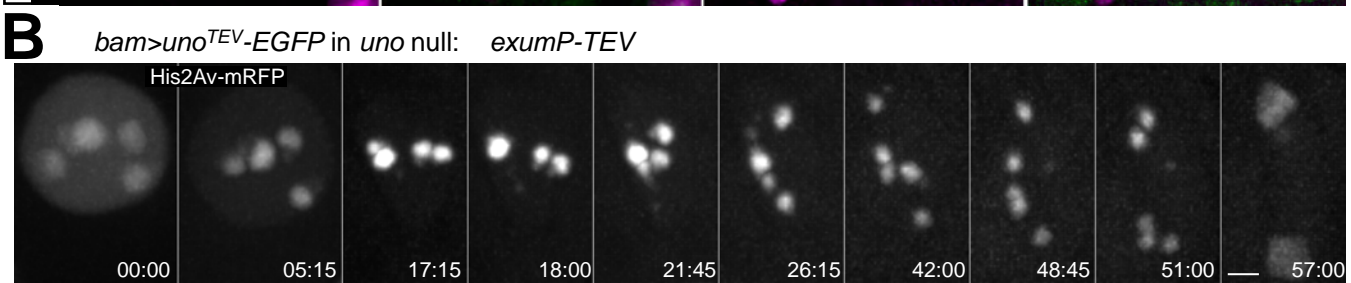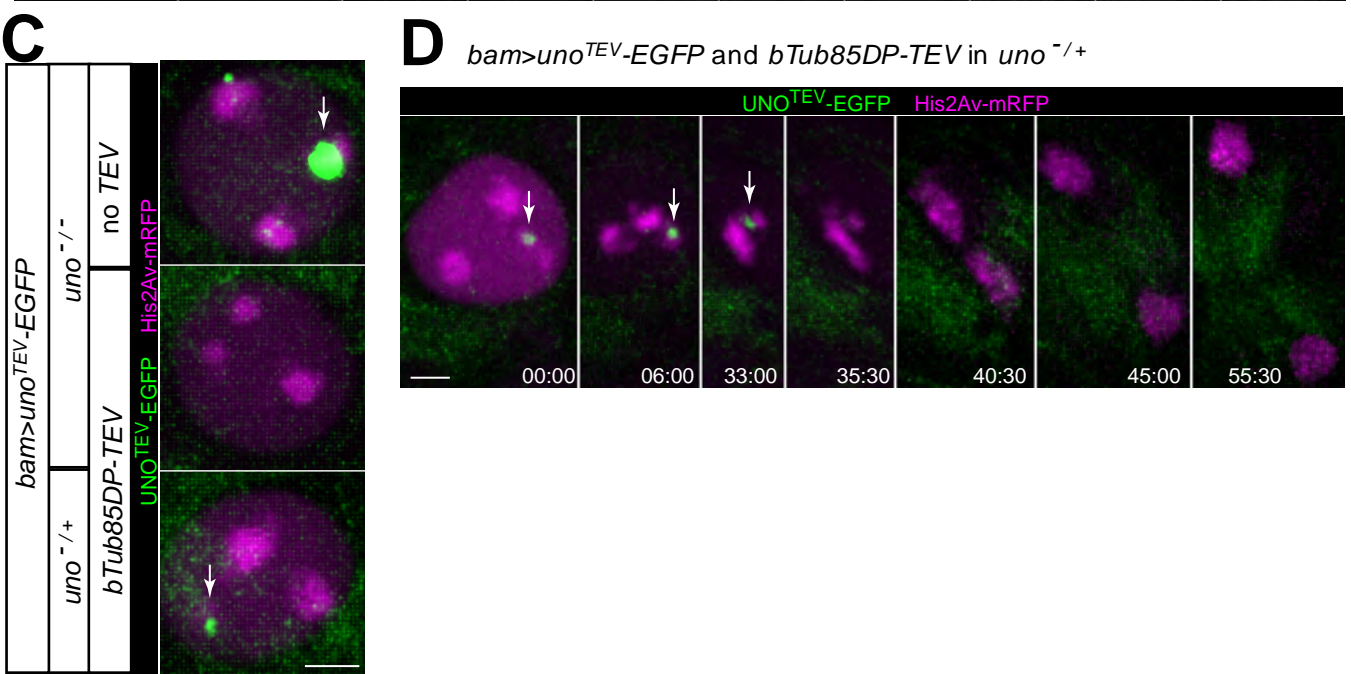

Supplement: S7 Fig — (A) Phenotypic characterization with squash preparations of testes from uno null mutants with bam>unoTEV-EGFP and either no TEV transgene or betaTub85DP-TEV (bTub85DP-TEV) provided further confirmation of the findings revealed by time-lapse imaging. In the absence of a TEV transgene UNOTEV-EGFP was readily detectable at the S6 stage with a sub-cellular localization identical to that of wild-type UNO-EGFP [22]. The S6 spermatocytes also displayed a normal number of major chromosome territories. However, in the presence of bTub85DP-TEV, UNOTEV-EGFP was not detectable in S6 spermatocytes, which displayed an increased number of chromosome territories. Each micrograph is shown twice, on the left with enhanced EGFP signal intensities, which reveal weak autosomal UNOTEV-EGFP dots in the absence of a TEV transgene and still no signals in the presence of bTub85DP-TEV. Still frames from cysts during telophase I analyzed by time-lapse imaging (bottom), illustrate the presence of massive chromosome bridges with persisting non-degradable UNOTEV-EGFP, but only in the absence of a TEV transgene. (B) Still frames after time-lapse imaging of His2A-mRFP expressing uno null mutants with bam>unoTEV-EGFP and exumP-TEV during M I reveal premature bivalent separation and absence of chromosome bridges during telophase I. Time (min:sec) with t = 0 at the onset of NEBD I is indicated. (C) Comparison of UNOTEV-EGFP signal intensities at the onset of NEBD I in indicated genotypes after time-lapse imaging. While UNOTEV-EGFP is completely eliminated by bTub85DP-TEV in unocc1 homozygous null mutant spermatocytes (uno-/-), residual UNOTEV-EGFP is detectable in unocc1 heterozygous spermatocytes (uno-/+) despite the presence of bTub85DP-TEV. (D) Disappearance of residual UNOTEV-EGFP (arrows) during anaphase I and absence of chromosome bridges during telophase I in uno-/+ spermatocytes with bam>unoTEV-EGFP and bTub85DP-TEV, as revealed by time-lapse imaging of spermatocytes expressing His2Av-mRFP. Ti [file pgen.1010547.s007.pdf]

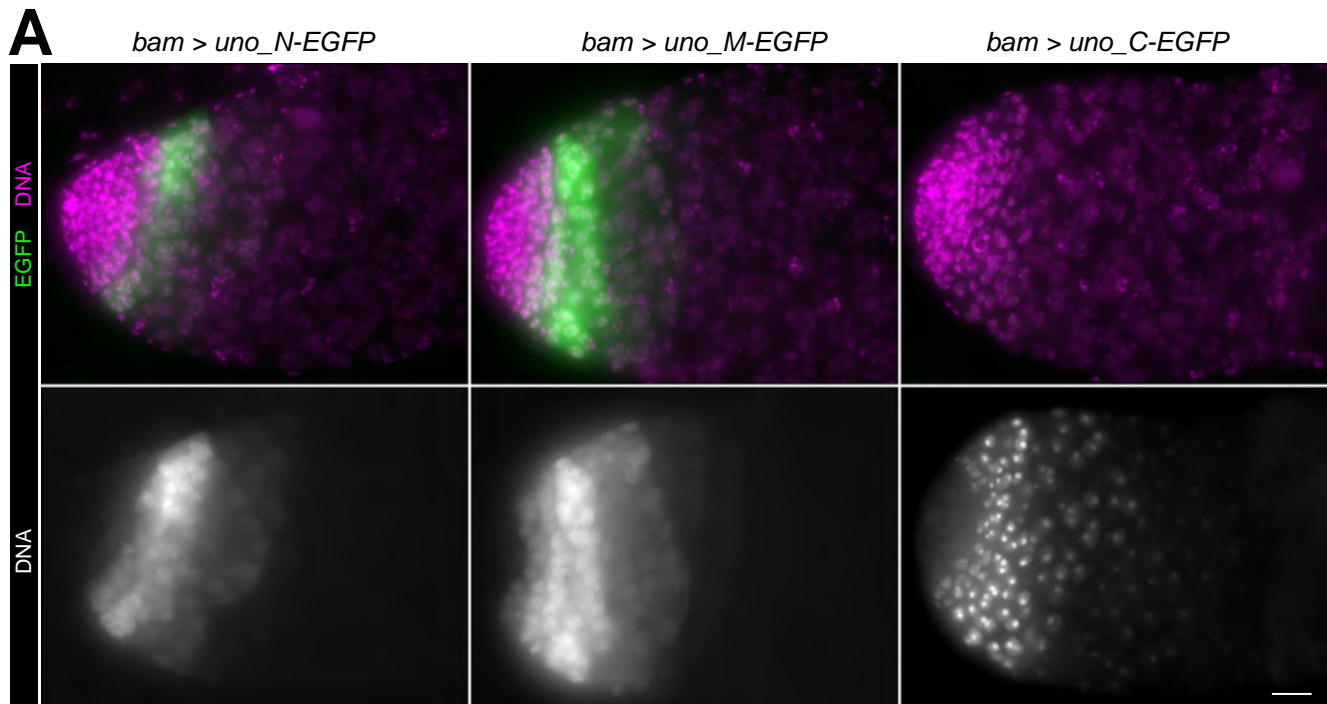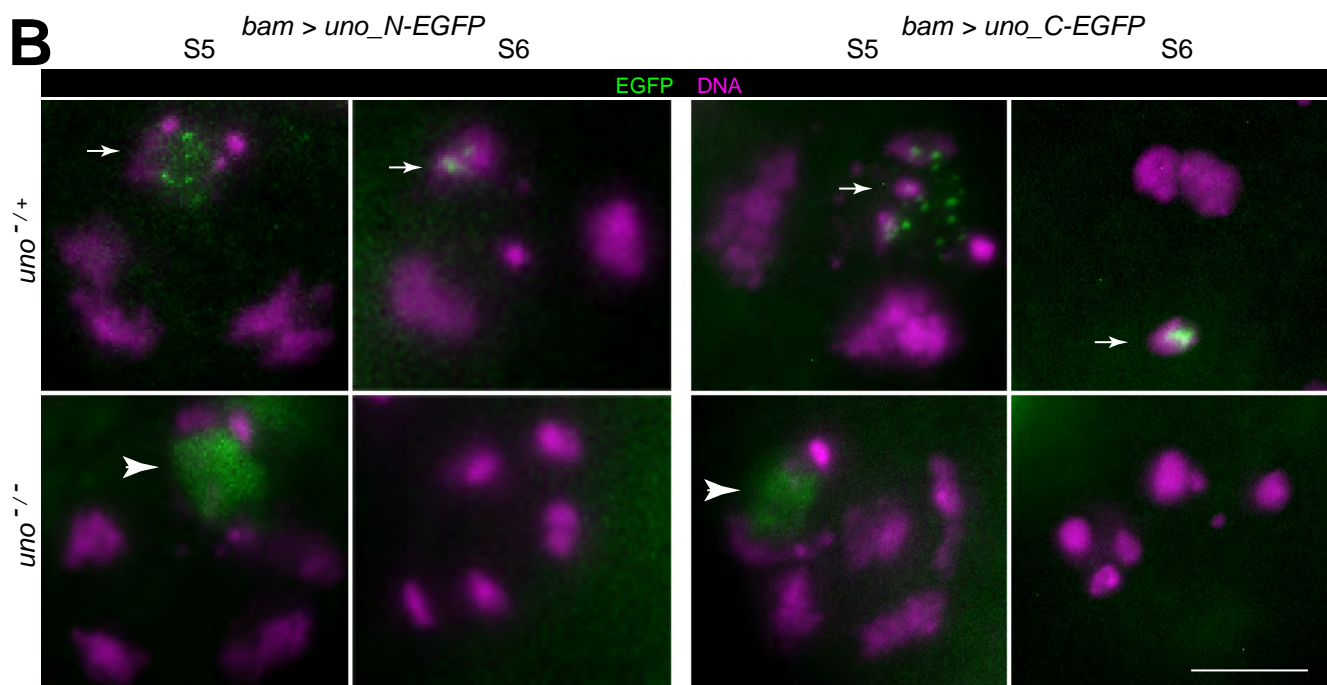

Supplement: S8 Fig — (A) The driver bamP-GAL4-VP16 was used for expression of the indicated UASt transgenes in an uno+ background. Testis squash preparations for comparison of expression level and pattern were labeled with a DNA stain. Apical testes regions are displayed. The images presenting merged DNA and EGFP channels (top row) are shown with identical settings during acquisition and display, indicating that expression levels were maximal in case of UNO_M-EGFP, weaker for UNO_N-EGFP and considerably weaker for UNO_C-EGFP. In case of the images displaying only the EGFP signals in grey values (bottom row), display settings were enhanced in case of UNO_C-EGFP to reveal its weak expression. (B) After peak expression of UNO_N-, UNO_M- and UNO_C-EGFP in early spermatocytes (see panel A), maintenance at low levels in late spermatocytes was detectable in case of UNO_N-EGFP and UNO_C-EGFP, as documented by high resolution of images from testes squash preparations. While at the S5 stage, UNO_N-EGFP and UNO_C-EGFP were in sub-nucleolar foci in spermatocytes heterozygous for the unocc1 null allele (uno-/+) (comparable to full-length UNO-EGFP, see Fig 5F), they displayed an abnormal diffuse nucleolar localization in unocc1 homozygous spermatocytes (uno-/-). At the S6 stage, UNO_N-EGFP and UNO_C-EGFP were in weak dots on the sex chromosome bivalent in uno-/+ spermatocytes and not detectable in uno-/- spermatocytes. Scale bars = 20 (A) and 5 (B) μm. (PDF) [file pgen.1010547.s008.pdf]

# A

*bam* > *uno*<sup>T128A</sup>-EGFP in *uno* null

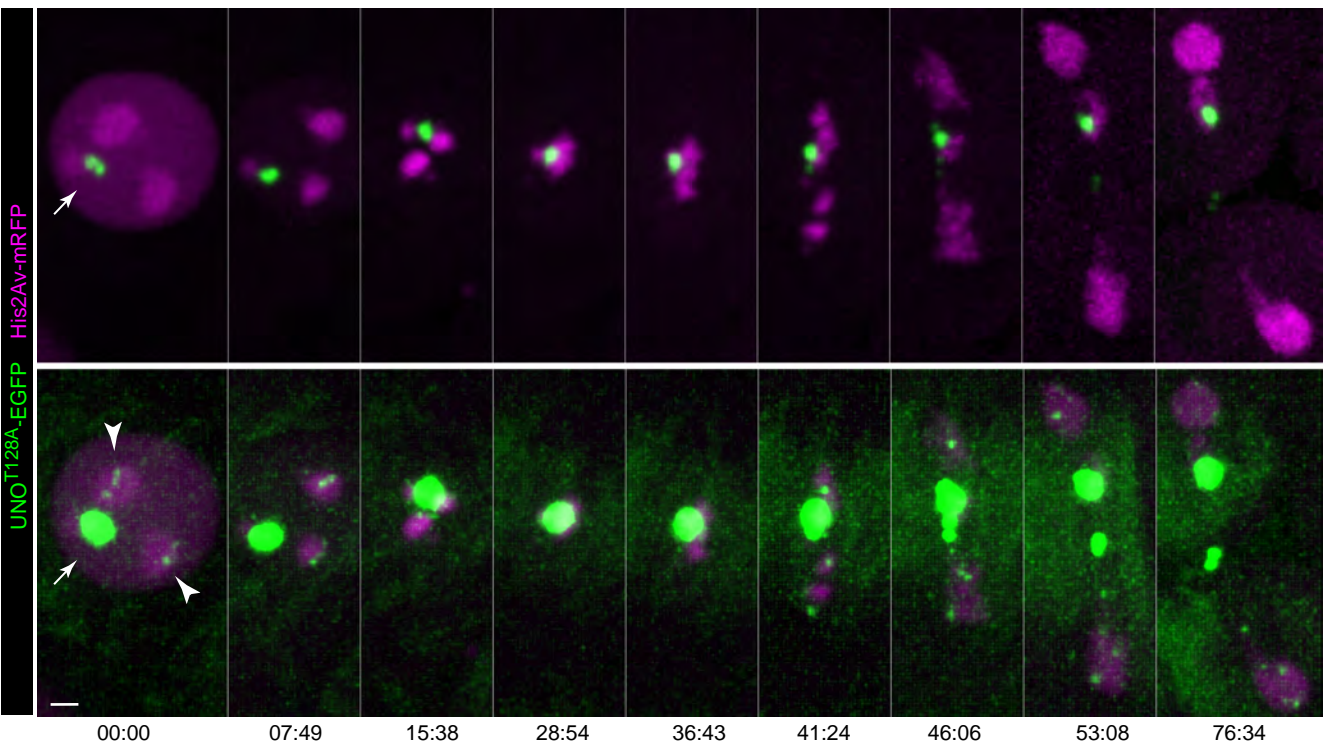

# B

*bam* > *uno*<sup>T128D</sup>-EGFP in *uno* null

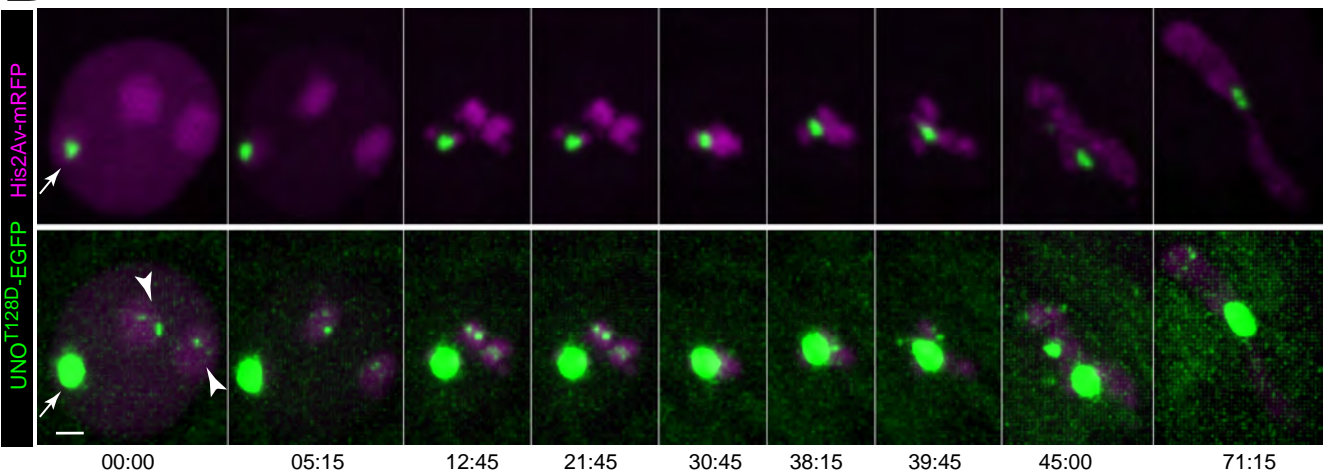

Supplement: S9 Fig — (A,B) Time-lapse imaging of progression through M I with His2Av-mRFP expressing spermatocytes was performed for the characterization of phenotypic consequences of the T128A (A) and T128 (B) mutations that alter a conserved potential phosphorylation site immediately upstream of the separase cleavage site in UNO (see Fig 7A). The mutants were expressed in uno null mutant spermatocytes with UASt transgenes and the driver bamP-GAL4-VP16. Time (min:sec) with t = 0 at the onset of NEBD I is indicated. Scale bars = 2 μm. (PDF) [file pgen.1010547.s009.pdf]
