# Supplementary material for: Homologous chromosomes are stably conjoined for Drosophila male meiosis I by SUM, a multimerized protein assembly with modules for DNA-binding and for separase-mediated dissociation co-opted from cohesin
Source: PLoS Genet. 2022 Dec 8;18(12):e1010547. doi: 10.1371/journal.pgen.1010547 (PMC9767379; doi:10.1371/journal.pgen.1010547)
Supplement: S2 Fig — An alignment of the predicted amino acid sequences of the indicated proteins reveals similarities between a region close to the C-terminus of UNO and an internal α-kleisin region, which is known to bind to stromalin/SA/STAG proteins. The family of α-kleisins includes members that function in meiosis (Rec8 proteins) beyond those functioning also during mitosis (Rad21 and Rad21L proteins). While the genome of D. melanogaster does not contain a canonical rec8 ortholog, it includes c(2)M, a more distant meiotic α-kleisin [79]. Accession numbers from the UniProt database (Dmel_uno, Dere_uno, Dpse_uno, Mdom_uno) or the NCBI database (all others) are provided next to the gene names with species abbreviated: Drosophila melanogaster (Dmel), Drosophila erecta (Dere), Drosophila pseudoobscura (Dpse), Musca domestica (Mdom), Ceratitis capitata (Ccap), Bactrocera dorsalis (Bdor), Homo sapiens (Hsap), Mus musculus (Mmus). Residues (triangles) of the human α-kleisin Rad21 that directly interact with human SA1 [36], as well as the α-helices (magenta rods) in Rad21 are indicated below the alignment. (PDF) [file pgen.1010547.s002.pdf]

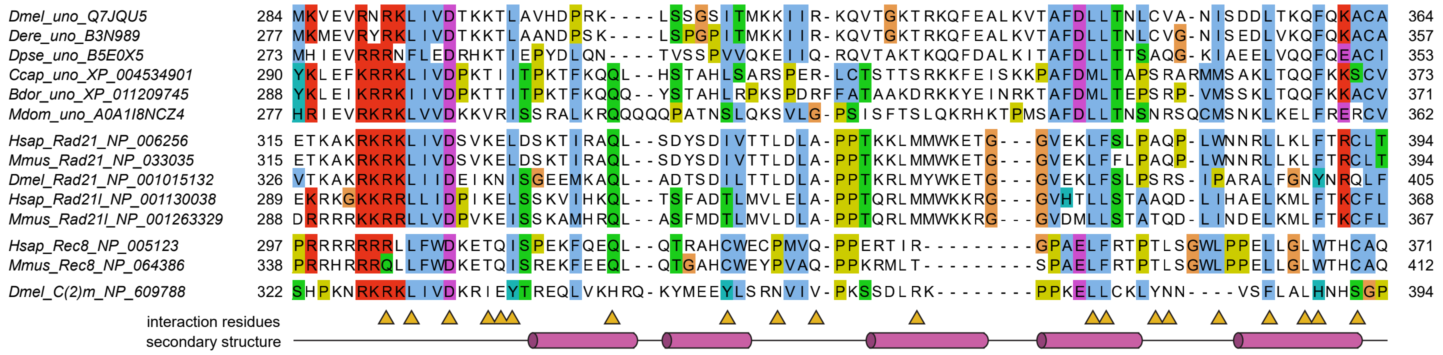

Dmel_uno_Q7JQU5 284 MKVEVRNRKLIVDTKKTLAVHDPRK----LSSGSITMKKIIR-KQVTGKTRKQFEALKVTAFDLLTNLCVA-NISDDLTKQFQKACA 364
Dere_uno_B3N989 277 MKMEVRYRKLIVDTKKTLAANDPSK----LSPGPITMKKIIR-KQVTGKTRKQFEALKVTAFDLLTNLCVG-NISEDLVKQFQKACA 357
Dpse_uno_B5E0X5 273 MHIEVRRRNFLEDRHKTIEPYDLQN----TVSSPVVQKEIIQ-RQVTAKTKQQFDALKITAFDLLTTSAQG-KIAEELVQQFQEACI 353
Ccap_uno_XP_004534901 290 YKLEFKRRKLIVDPKTIITPKTFKQQL--HSTAHLSARSPER-LCTSTTSRKKFEISKKPAFDMLTAPSRARMMSAKLTQQFKKSCV 373
Bdor_uno_XP_011209745 288 YKLEIKRRKIIVDPKTTITPKTFKQQQ--YSTAHLRPKSPDRFFATAAKDRKKYEINRKTAFDMLTEPSRP-VMSSKLTQQFKKACV 371
Mdom_uno_A0A1I8NCZ4 277 HRIEVRKRKLVVDKKVRISSRALKRQQQQQPATNSLQKSVLG-PSISFTSLQKRHKTPMSAFDLLTNSNRSQCMSNKLKELFRERCV 362
Hsap_Rad21_NP_006256 315 ETKAKRKRKLIVDSVKELDSKTIRAQL--SDYSDIVTTLDLA-PPTKKLMMWKETG---GVEKLFSLPAQP-LWNNRLLKLFTRCLT 394
Mmus_Rad21_NP_033035 315 ETKAKRKRKLIVDSVKELDSKTIRAQL--SDYSDIVTTLDLA-PPTKKLMMWKETG---GVEKLFFLPAQP-LWNNRLLKLFTRCLT 394
Dmel_Rad21_NP_001015132 326 VTKAKRKRKLIIDEIKNISGEEMKAQL--ADTSDILTTLDLA-PPTKRLMYWKETG---GVEKLFSLPSRS-IPARALFGNYNRQLF 405
Hsap_Rad21l_NP_001130038 289 EKRKGKKRRLLIDPIKELSSKVIHKQL--TSFADTLMVLELA-PPTQRLMMWKKRG---GVHTLLSTAAQD-LIHAELKMLFTKCFL 368
Mmus_Rad21l_NP_001263329 288 DRRRRKKRRLLVDPVKEISSKAMHRQL--ASFMDTLMVLDLA-PPTQRLMMWKKRG---GVDMLLSTATQD-LINDELKMLFTKCFL 367
Hsap_Rec8_NP_005123 297 PRRRRRRRLLFWDKETQISPEKFQEQL--QTRAHCWECPMVQ-PPERTIR---------GPAELFRTPTLSGWLPPELLGLWTHCAQ 371
Mmus_Rec8_NP_064386 338 PRRHRRRQLLFWDKETQISREKFEEQL--QTGAHCWEYPVAQ-PPKRMLT---------SPAELFRTPTLSGWLPPELLGLWTHCAQ 412
Dmel_C(2)m_NP_609788 322 SHPKNRKRKLIVDKRIEYTREQLVKHRQ-KYMEEYLSRNVIV-PKSSDLRK--------PPKELLCKLYNN----VSFLALHNHSGP 394
interaction residues
secondary structure
