# Supplementary material for: Homologous chromosomes are stably conjoined for Drosophila male meiosis I by SUM, a multimerized protein assembly with modules for DNA-binding and for separase-mediated dissociation co-opted from cohesin
Source: PLoS Genet. 2022 Dec 8;18(12):e1010547. doi: 10.1371/journal.pgen.1010547 (PMC9767379; doi:10.1371/journal.pgen.1010547)
Supplement: S6 Fig — FRAP analyses were completed with S5 cysts released from pupal testes of males expressing the indicated UASt transgenes driven by bam-GAL4-VP16. A subregion of the nucleolus was bleached in one of the spermatocytes of the cyst, while neighboring spermatocytes were used as controls, as illustrated with the still frames acquired during an experiment with SNM-EGFP just before and after the bleaching. The timeline on top illustrates the image acquisition sequence. EGFP signal intensities in the nucleoli were quantified and are plotted after normalization to the average of the intensities observed before bleaching. (PDF) [file pgen.1010547.s006.pdf]

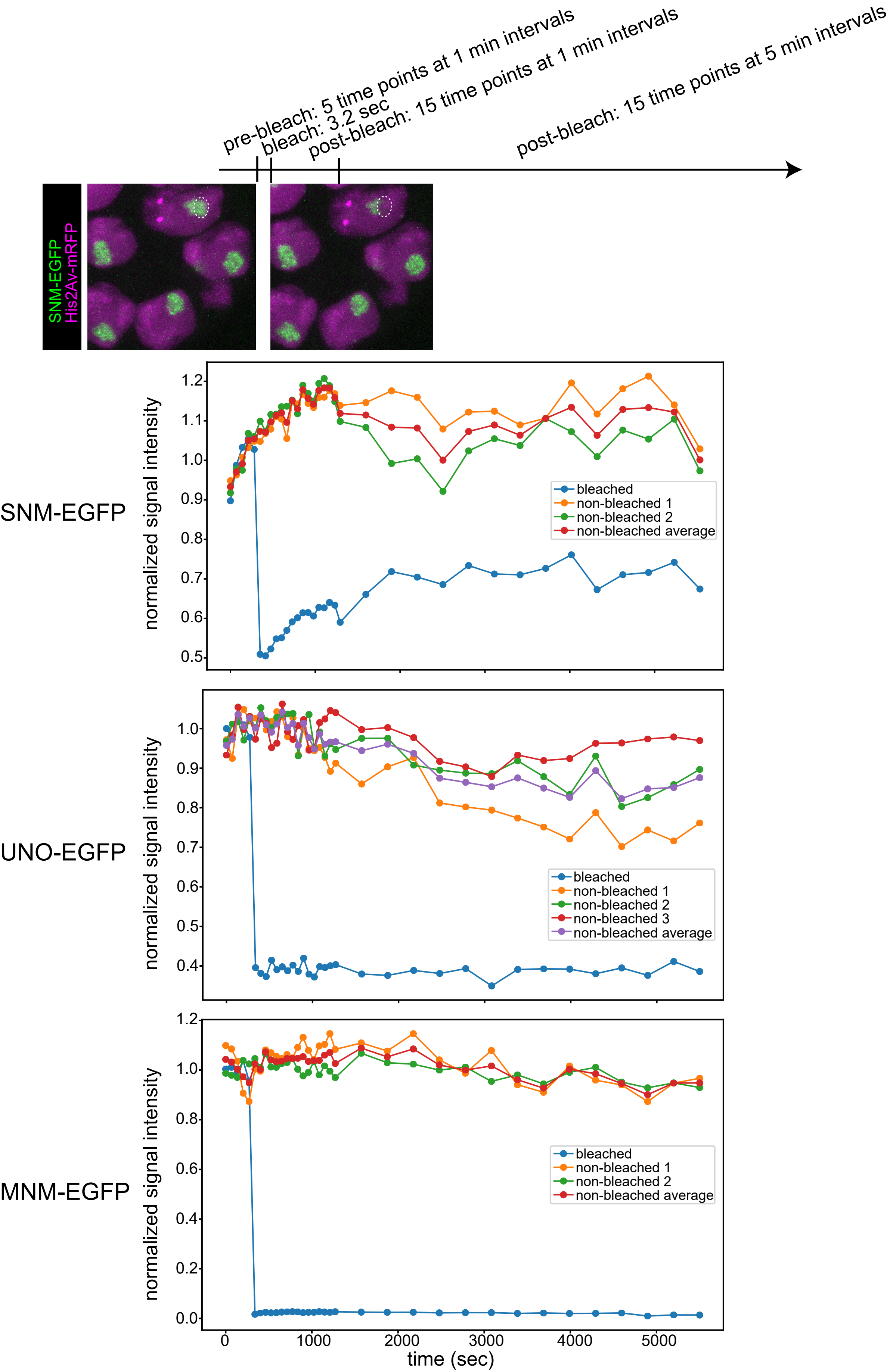
pre-bleach: 5 time points at 1 min intervals
bleach: 3.2 sec
post-bleach: 15 time points at 1 min intervals
post-bleach: 15 time points at 5 min intervals
SNM-EGFP
His2Av-mRFP
SNM-EGFP
UNO-EGFP
MNM-EGFP
normalized signal intensity
bleached
non-bleached 1
non-bleached 2
non-bleached 3
non-bleached average
time (sec)
