# Supplementary material for: Homologous chromosomes are stably conjoined for Drosophila male meiosis I by SUM, a multimerized protein assembly with modules for DNA-binding and for separase-mediated dissociation co-opted from cohesin
Source: PLoS Genet. 2022 Dec 8;18(12):e1010547. doi: 10.1371/journal.pgen.1010547 (PMC9767379; doi:10.1371/journal.pgen.1010547)
Supplement: Attachment — Submitted filename: Response to Reviewers.pdf [file pgen.1010547.s018.pdf]

Response to Reviewer #1:

We were very pleased to read the highly favorable comments, and appreciate the pointing out of the typo in line 1478, which we have corrected. The only additional suggestion for revision was that "It might be an interesting addition however, if the authors include ideas on if/how they envision the complex is restricted to homologs rather than making connections between sister chromatids."

In response, we have added an additional paragraph into the discussion (lines 623-641 in the revised version).

In connection with this additional paragraph, we would like to respond to another statement made by reviewer 1 when commenting on the significance of our work: "A unexplained aspect of this model [topological AHC by ring-shaped protein complexes around homologs], however, was how mutants in sister chromatid cohesion proteins such as *solo* and *sunn* result in precocious separation of sister chromatids prior to Anaphase I. It was difficult to imagine how a ring surrounding homologs would not perforce bind sisters together as well." We point out that the precocious separation of sister chromatids prior to anaphase I in *solo* and *sunn* is restricted to sister centromeres and pericentromeric region in spermatocytes (Yan et al. (McKee) 2010. JCB; Krishnan et al. (McKee) 2015. Genetics). The precocious sister chromatid separation is incomplete in spermatocytes. Moreover, it is likely that the AHC proteins preclude the complete precocious sister chromatid separation in these single mutant spermatocytes, because in *sunn snm* double mutants, there is a complete precocious sister chromatid separation before anaphase I (Yan et al. (McKee) 2010.JCB).

Response to Reviewer #2:

This reviewer had little criticism except for the five points below to which we respond as follows:

1) Figures 1 and 2 are pretty dense, but I also don't have a good suggestion on how to improve them.

Unfortunately, we also see no solution that would make the figures less dense.

2) Line 90-91: it may be worth being more specific that centromeric cohesion depends on protecting meiotic kleisins like Rec8 with proteins like MEI-S332 and DMT that recruit PP2A.

As protection of centromeric cohesion is of peripheral importance for our work on AHC, we consider the insertion of these additional mechanistic details into the introduction to be more distractive than helpful. The references given in the preceding sentence will provide such details for readers interested in the current understanding of the molecular mechanisms.

3) Line 290: the computational prediction looks like a good working model; "correct" is an overstatement.

In the revised version we state: …., thus suggesting that the SU_C structure model is likely a good fit (line 290 in the revised version).

4) Pg 15: the DNA content assay on this page for chromosome segregation is crude. There are much better and accurate cytological assays, including some previously published by this PI.

In addition to the DNA content assay, our manuscript also includes an analysis by time lapse imaging. Thus, we consider the analysis of the phenotypic consequences of the expression of UNO[chm]-EGFP instead of wild-type UNO during M I to be sufficiently solid to justify our conclusions.

5) The results in Figure 7 are not very surprising. If there was a place to shorten the paper, it would be here. The reason is that it was already known that UNO was a target of separase mutating the cleavage site resulted in defects in homolog separation. Replacing the separase site with a TEV site is essentially the same experiment. Then adding TEV protease only shows what happens when UNO is degraded. It is not a biologically important results because degrading UNO by any number of

methods would result in the same phenotype. For example, targeting SNM with TEV might have the same effect.

We agree that the replacement of the separase site in UNO with the TEV cleavage site and observing a failure of homolog separation during M I in the absence of TEV expression is largely equivalent to our earlier analyses of the effect of point mutations within the separase cleavage site that precluded cleavage by separase. However, we disagree that "adding TEV expression only shows what happens when UNO is degraded." TEV is a site-specific protease. It cleaves UNO into two fragments. It does not degrade UNO like the proteasome. Moreover, TEV is expressed after the formation of chromosomal SUM protein assemblies that are stable according to our FRAP experiments. Thus, we are not just making an *uno* null mutant in a complicated way. While targeting SNM with TEV might perhaps result in the same phenotype, but depending on the position of the TEV site within SNM it also might not. Targeting SNM with TEV will likely not have the same effect if the TEV cleavage site is positioned into a surface loop that is not required for maintenance of the folded conformation and if TEV is expressed after the chromosomal SUM protein assemblies have been formed already (as in our experiments). The absence of chromosomal UNO[TEV]-EGFP dots after TEV expression does not necessarily indicate that the UNO[TEV]-EGFP fragments resulting from TEV cleavage are further degraded. It could also reflect re-distribution of the UNO[TEV]-EGFP fragments throughout the cell, resulting in diffuse signals no longer detectable above background. However, most importantly, our preceding experiments do not rule out the possibility that separase might need to cleave additional targets beyond UNO to permit homolog separation. The results of our TEV experiment argue strongly against this possibility.
