# Supplementary material for: Homologous chromosomes are stably conjoined for Drosophila male meiosis I by SUM, a multimerized protein assembly with modules for DNA-binding and for separase-mediated dissociation co-opted from cohesin
Source: PLoS Genet. 2022 Dec 8;18(12):e1010547. doi: 10.1371/journal.pgen.1010547 (PMC9767379; doi:10.1371/journal.pgen.1010547)
Supplement: Attachment — Submitted filename: Review PGenetics.docx [file pgen.1010547.s017.docx]

PGENETICS-D-22-01170

Homologous chromosomes are stably conjoined for *Drosophila* male meiosis I by SUM, a multimerized protein assembly with modules for DNA-binding and for separase-mediated dissociation co-opted from cohesion.

Zeynep Kabakci , Heidi E. Reichle , Bianca Lemke , Dorota Rousova, Samir Gupta , Joe Weber , Alexander Schleiffer , John R. Weir* and Christian F. Lehner*

This manuscript represents a substantial and significant contribution to our understanding of achiasmate homolog conjunction (AHC) in male Drosophila, an organism that has been studied as a model of alternative meiosis for almost a century. I feel that it will be of great interest to researchers in the field of meiosis, and to a much larger audience of geneticists and cell biologists in general.

Proteins involved in segregating homologs in the absence of recombination have been known for some time in Drosophila, but previous studies have provided limited information on the mechanisms by which these proteins interact to facilitate conjunction. In previous work, the Lehner lab has reported on the discovery and characterization of the “missing link” in this system, a protein they named UNO (UNivalents Only). This protein was found to have homology to alpha-kleisins, and in particular, to possess a functional separase cleavage site necessary for the separation of homologs at Anaphase I. These findings led to a model in which homologs were hypothesized to be conjoined by a ring structure analogous to that formed by cohesins. A unexplained aspect of this model, however, was how mutants in sister chromatid cohesion proteins such as *solo* and *sunn* result in precocious separation of sister chromatids prior to Anaphase I. It was difficult to imagine how a ring surrounding homologs would not perforce bind sisters together as well.

In this manuscript, Kabakci et al. exhaustively examine physical interactions between components of the AHC complex (SNM, UNO, MNM and TEF) by a variety of methodologies, and propose a convincing new model of how this SUM complex mediates conjunction, involving multimerization of UNO and MNM subunits. They propose that SUM acts effectively as a glue between homologs, rather than an encompassing ring, that is bound together by connection to the separase-cleavable UNO subunits.

Pulldowns using purified proteins establish domains of each protein that interact, and the data in each case are carefully controlled and convincing. Interestingly, they show that MNM can alternatively complex with TEF or SNM/UNO, but these interactions are mutually exclusive. Assembly of the SUM complex, however, allows for multiple connections, thus allowing the complex involving multimers of MNM to simultaneously bind to TEF. They also show by EMSA that a SNM/UNO complex has DNA binding activity, but somewhat surprisingly does not show specificity for the 240 bp intergenic spacer DNA known to be the main site of conjunction of the sex chromosomes.

In additional to pulldowns, they demonstrate SNM/UNO complex formation by sepharose chromatography, and employ computer modeling to guide site-directed mutagenesis to test for critical residues in complex formation and DNA binding. Using FRAP, they demonstrate that the SUM complex is stable once assembled, and shows little turnover. To demonstrate the importance of UNO cleavage in releasing the complex at anaphase, they perform a clever experiment in which they substitute the separase cleavage site for a TEV cleavage site in UNO, and show that homolog separation then becomes TEV-dependent. Despite their inability to express and assemble in vitro purified full length proteins of the SUM complex, overall, this is truly a tour de force analysis.

The quality of the data and style which it is presented for each experiment is exceptional. The manuscript is very clearly written, each experiment is explained in sufficient detail, and the images are clear, convincing and well-organized. I have no substantial criticisms of the data or its interpretations. It might be an interesting addition however, if the authors include ideas on if/how they envision the complex is restricted to homologs rather than making connections between sister chromatids.

Minor correction : typo on line 1478
